# COVID-19: Coronavirus Vaccine Development Updates

**DOI:** 10.3389/fimmu.2020.602256

**Published:** 2020-12-23

**Authors:** Jing Zhao, Shan Zhao, Junxian Ou, Jing Zhang, Wendong Lan, Wenyi Guan, Xiaowei Wu, Yuqian Yan, Wei Zhao, Jianguo Wu, James Chodosh, Qiwei Zhang

**Affiliations:** ^1^ Guangdong Provincial Key Laboratory of Tropical Disease Research, School of Public Health, Southern Medical University, Guangzhou, China; ^2^ Guangdong Provincial Key Laboratory of Virology, Institute of Medical Microbiology, Jinan University, Guangzhou, China; ^3^ Department of Ophthalmology, Howe Laboratory, Massachusetts Eye and Ear, Harvard Medical School, Boston, MA, United States

**Keywords:** Severe Acute Respiratory Syndrome, vaccine, Coronavirus Disease 2019 (COVID-19), Severe Acute Respiratory Syndrome Coronavirus 2, Middle-East Respiratory Syndrome

## Abstract

Coronavirus Disease 2019 (COVID-19) is caused by Severe Acute Respiratory Syndrome Coronavirus 2 (SARS-CoV-2), a newly emerged coronavirus, and has been pandemic since March 2020 and led to many fatalities. Vaccines represent the most efficient means to control and stop the pandemic of COVID-19. However, currently there is no effective COVID-19 vaccine approved to use worldwide except for two human adenovirus vector vaccines, three inactivated vaccines, and one peptide vaccine for early or limited use in China and Russia. Safe and effective vaccines against COVID-19 are in urgent need. Researchers around the world are developing 213 COVID-19 candidate vaccines, among which 44 are in human trials. In this review, we summarize and analyze vaccine progress against SARS-CoV, Middle-East respiratory syndrome Coronavirus (MERS-CoV), and SARS-CoV-2, including inactivated vaccines, live attenuated vaccines, subunit vaccines, virus like particles, nucleic acid vaccines, and viral vector vaccines. As SARS-CoV-2, SARS-CoV, and MERS-CoV share the common genus, *Betacoronavirus*, this review of the major research progress will provide a reference and new insights into the COVID-19 vaccine design and development.

## Introduction

Coronaviruses are members of the subfamily *Coronavirinae* composed of four genera -*Alphacoronavirus*, *Betacoronavirus*, *Gammacoronavirus*, and *Deltacoronavirus*, in the family *Coronaviridae*, under the order *Nidovirales* (1). Coronaviruses are positive sense, single-stranded RNA viruses with a spherical shape envelope, a diameter of 100–160 nm and a genome size of 27–32 kb. The 5’ end of the genome occupies approximately 2/3 of the total length and encodes polyprotein (pp1ab), which is cleaved to 16 non-structural proteins involved in the transcription and replication of the genome. The 3’ end encodes structural proteins, including envelope spike glycoproteins (S), envelope (E), membrane glycoprotein (M), and nucleocapsid (N) (1). S1 subunit of the spike glycoprotein mediates recognition by host receptors and S2 subunit promotes fusion of viral envelope with the cell membrane. E and M proteins are responsible for the transmembrane transport assembly, budding, and release of progeny viruses, and the formation of virus envelopes, all of which play an important role in virus production and maturity (2). N protein binds to viral RNA, which is involved in the viral gene replication cycle and immune response to viral infections of host cells (2, 3). Otherwise, there are species-specific accessory genes which are also essential for viral replication (1).

There are seven types of coronaviruses relevant to humans, four of which are human coronaviruses (HCoV-NL63, HCoV-229E, HCoV-OC43, and HKU1), causing limited mild upper respiratory symptoms in immunocompetent populations, while the other three are highly pathogenic coronaviruses - Severe Acute Respiratory Syndrome Coronavirus (SARS-CoV), Middle East Respiratory Syndrome Coronavirus (MERS-CoV) and novel Coronavirus (SARS-CoV-2), all causing severe respiratory disease in humans.

The symptoms of SARS usually include fever, chills, and body pain, and infection can develop into pneumonia. According to WHO statistics, from November 1, 2002 to July 1 2003, 8,096 cases and 774 deaths had been confirmed with SARS-CoV infection worldwide, with a fatality rate of 9.6%. MERS is a viral respiratory disease caused by MERS-CoV and was first confirmed in Saudi Arabia in 2012. MERS symptoms usually include fever, cough, and shortness of breath, and infection can also lead to pneumonia. Since 2012, MERS has spread to 27 countries and regions in the Middle East, Asia, and Europe (4), and 80% of cases are from Saudi Arabia. 2,494 cases and 858 deaths with MERS-CoV infection have been reported, with a fatality rate of about 35%. The incubation period is up to 14 days, and the world population is generally susceptible. Dromedary camels are a major host of MERS-CoV and are the main source of infection of humans, with only limited human-to-human transmission.

In December 2019, SARS-CoV-2, a novel coronavirus was identified in Wuhan, China as a new Betacoronavirus (5). This new virus causes Coronavirus Disease 2019 (COVID-19). On February 28th, WHO declared the global emergency risk level as “very high”. On March 12th, the global COVID-19 outbreaks were declared as a pandemic. Many cities around the world mandated lockdowns. As of November 1st, 2020, the pandemic had caused 45,968,799 confirmed cases and 1,192,911 fatalities with the estimated case fatality rate of 2.60% (https://covid19.who.int/). Comparative genomic analysis showed the divergence of SARS-CoV-2 and identified 380 amino acid substitutions between SARS-CoV-2 (Wuhan/HB01 strain) and SARS-CoV (6).

Currently, the pandemic of COVID-19 is still evolving. Effective therapeutic drugs for severe cases and effective vaccines for the healthy people are in urgent need. However, there is no specific prescription drug or effective vaccine licensed to treat or prevent COVID-19 worldwide except for four vaccines for limited use in China and two vaccines for early use in Russia.

**Figure 1:**
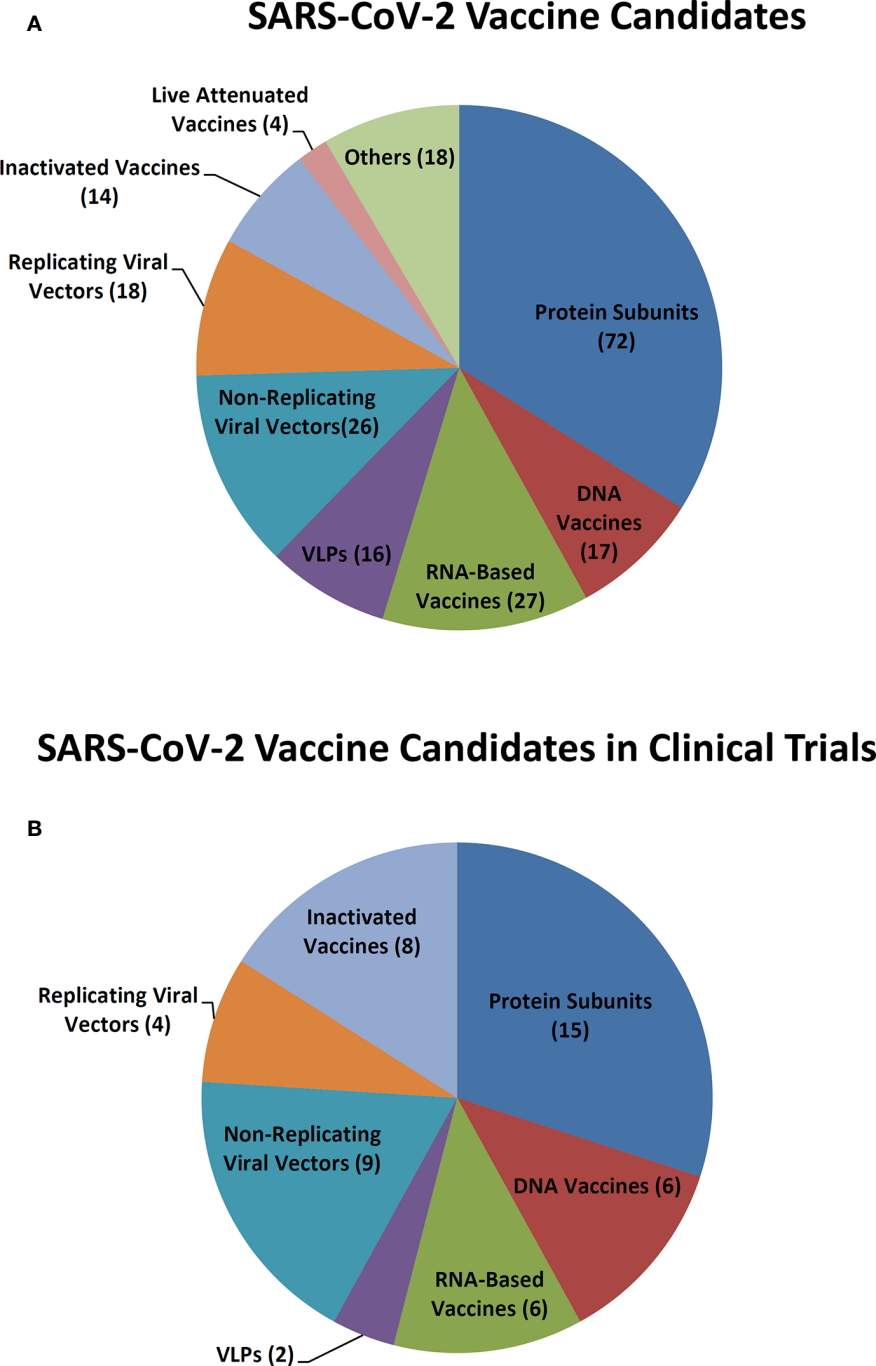
SARS-CoV-2 vaccine candidates. **(A)** SARS-CoV-2 vaccine candidates in development. **(B)** SARS-CoV-2 vaccine candidates in clinical trials.

As of October 19, 2020, among 212 SARS-CoV-2 candidate vaccines being developed all over the world, 50 have been under clinical evaluation and 162 are in preclinical development (**Figure 1**). Among them, there are 14 inactivated vaccines, four live attenuated vaccines, 72 protein subunit vaccines, 17 DNA vaccines, 27 RNA-based vaccines, 16 virus-like particle (VLP) vaccines, 26 non-replicating viral vector vaccines, and 18 replicating viral vector vaccines (**Figure 1A**). For the vaccines in clinical trials, eight are inactivated vaccines, 15 are protein subunit vaccines, six are DNA vaccines, six are RNA-based vaccines, two are VLPs vaccines, nine are non-replicating viral vector vaccines, and four are replicating viral vector vaccines (**Figure 1B**).

In China, there are 13 vaccine candidates that have entered clinical trials, of which six vaccine candidates are currently in phase III clinical trials. As SARS-CoV-2 is similar to the highly pathogenic SARS-CoV and MERS-CoV, experiences in the development of vaccines against other *Betacoronaviruses* may facilitate the COVID-19 vaccine development. In this study, we briefly review past and current CoV vaccine research and development against SARS (**Table 1**), MERS (**Table 2**), and SARS-CoV-2 (**Table 3**) (**Figure 1**), including inactivated vaccines, live attenuated vaccines, subunit vaccines, virus like particles, nucleic acid vaccines, and viral vector vaccines, aiming to provide a reference and new insights, to facilitate the better and faster development of COVID-19 vaccines.

**Table 1:** SARS-CoV vaccine candidates.

Vaccine type	Adjuvant	Animal model	Humoral immunity	Cellular immunity	Protective immunity	Reference
RBD-Fc	MF59, Alum, etc.	129S6/SvEv mice	+	+	+	(7–15)
N protein		BALB/C mice	+	+	+	(16–19)
Recombinant VLPs-S(VLPs)	Alum	BALB/c mice	+	+	+	(20–25)
DNA-S		BALB/c mouse, human	+	+	+	(26, 27)
DNA-N (pVAXN)		BALB/c mice	+	+		(28)
MVA-S		BALB/c mice, New Zealand white rabbits, NHPs	+		+	(29–32)
AdV-S		BALB/c mice	+	+	+	(33–35)
MV-S		CD46-IFNAR mice	+		+	(36, 37)
Inactivated vaccine	δ- inulin	mice	+	+	+	(38)
UV-inactivated vaccine	TLR-3	BALB/c mice	+		+	(39)
Inactivated vaccine SARS-CoV Z-1		NHPs	+		+	(40)
Live attenuated vaccine SARS-CoV-ΔE		BALB/c mice	+	+	+	(41, 42)
Live attenuated vaccine SARS-dE-CTD&-NTD d8-12aa		BALB/c Ola Hsd mice	+	+	+	(43)
Live attenuated vaccine nsp16 mutant		BALB/c mice	+		+	(44)
Live attenuated vaccine SARS-CoV-ExoN(-)		SCID mice	+		+	(45, 46)

Protective immunity: prevention of SARS-CoV infection in virus challenged animals, lower viral titers with mild or without histopathological changes in lungs, including inflammation of bronchial epithelium and damage of alveolar walls, eosinophilic infiltration in lung tissue, etc.

**Table 2:** MERS-CoV vaccine candidates.

Vaccine type	Adjuvant	Animal model	Humoral immunity	Cellular immunity	Protective immunity	Reference
RBD-Fc	MF59, Alum, etc.	hDPP4-Tg BALB/c mice	+	+	+	(47–49)
RBD trimer	Alum	Mice	+			(50)
Nanoparticles(S)	Matrix M1	hDPP4-Tg BALB/c mice	+		+	(51)
S prefusion trimer	Sigma adjuvant	BALB/cJ mice	+		+	(52)
S1-NTD		hDPP4-tgmice	+	+	+	(53, 54)
Recombinant VLPs (pFastBacDual-M1-St/Hak)	Alum	BALB/c mice	+	+		(23)
BLPs (RLP3-GEM)	GEL01	BALB/c mice	+	+	+	(55)
S DNA (pVax1TM-S) (GLS-5300)		NHPs, Human	+	+	+	(56, 57)
S DNA (pVRC8400)	AlPO_4_	BALB/cJ mice, NHPs	+		+	(58, 59)
DNA+S(S1 enhancement)		NHPs, Mice	+		+	(58)
DNA-S1(pcDNATM3.1 (+)-S1)		Mice	+	+	+	(60, 61)
MVA-S		BALB/c mice, NHPs	+	+	+	(62–65)
Ad5-S/S1		BALB/c mice	+			(34)
Ad5-S		BALB/c mice	+	+		(33)
Ad41-S		BALB/c mice	+	+		(33)
Ad5-S+S nanoparticle		AdV-hDPP4-Tg BALB/c mice		+	+	(66)
ChAdOx1-S		BALB/c mice, NHPs	+	+	+	(67–69)
AdC68-S		hDPP4-KI BALB/c mice	+	+	+	(70)

Protective immunity: prevention of MERS-CoV infection in virus challenged animals, lower viral titers with mild or without histopathological changes in lungs, including inflammation of bronchial epithelium and damage of alveolar walls, eosinophilic infiltration in lung tissue, etc.

**Table 3:** SARS-CoV-2 vaccines in clinical phase III trials and early or limited use.

Vaccine Candidates	Developer	Country	Vaccine Platform	Allocation & Masking	Doses	Timing of doses	Route of Administration	No.
	Wuhan Institute Of Biological Products/Sinopharm	China	Inactivated	Randomized, Double Blind, Parallel Placebo Controlled	2	0, 21days	IM	ChiCTR2000031809 ChiCTR2000034780 ChiCTR2000039000
BBIBP-CorV	Beijing Institute Of Biological Products/Sinopharm	China	Inactivated	Randomized, Triple Blind(Participant, Care Provider, Investigator), Parallel Placebo Controlled	2	0, 21days	IM	ChiCTR2000032459 ChiCTR2000034780 NCT04560881
	Sinovac/Instituto Butantan/Bio Farma	China, Brazil	Inactivated	Randomized, Quadruple Blind(Participant, Care Provider, Investigator, Outcomes Assessor), Parallel Placebo-Controlled	2	0, 14 days	IM	NCT04383574 NCT04352608 NCT04551547 NCT04456595 NCT04582344 669/UN6.KEP/EC/2020
Ad5-nCOV	Cansino Biologics/Beijing Institute Of Biotechnology/Canada’S National Research Council/Petrovax	China, Canada	Non-Replicating Viral Vector	Randomized, Quadruple Blind(Participant, Care Provider, Investigator, Outcomes Assessor), Parallel Placebo-controlled	1(5 × 10^10^ vp)		IM	ChiCTR2000030906 ChiCTR2000031781 NCT04313127 NCT04341389 NCT04398147 NCT04526990 NCT04540419 NCT04566770 NCT04568811
Sputnik V	Gamaleya Research Institute	Russia	Non-Replicating Viral Vector	Randomized, Double Blind(Participant, Investigator), Parallel Placebo-controlled	2 (0.5 ml/dose+0.5 ml/dose prime-boost)	0, 21days	IM	NCT04437875 NCT04436471 NCT04530396 NCT04564716 NCT04587219
EpiVacCorona	Federal Budgetary Research Institution (Fbri) State Research Center Of Virology And Biotechnology “Vector”	Russia	Protein Subunit	Randomized, Single Blind(Participant), Parallel Placebo-controlleds	2 (0.5 ml)	0, 21days	IM	NCT04527575
Ad26.COV2-S	Janssen Pharmaceutical Companies/Beth Israel Deaconess Medical Center/Emergent Biosolutions/Catalent/Biological E/Grand River Aseptic Manufacturing (Gram)	USA	Non-Replicating Viral Vector	Randomized, Quadruple Blind(Participant, Care Provider, Investigator, Outcomes Assessor), Parallel Placebo-controlled	2 (5 × 10^10^ vp)	0, 56days	IM	NCT04436276 NCT04505722
AZD 1222	University Of Oxford, Oxford Biomedica, Vaccines Manufacturing And Innovation Centre, Pall Life Sciences, Cobra Biologics, Halixbv, Advent S.R.L., Merck Kgaa, The Serum Institute, Vaccitech, Catalent, Csl, And Astrazeneca/Iqvia	UK	Non-Replicating Viral Vector	Randomized, Quadruple Blind(Participant, Care Provider, Investigator, Outcomes Assessor), Parallel Placebo-controlled	2 (5 × 10^10^ virus particles (vp))	0, 28days	IM	CTRI/2020/08/027170 EudraCT 2020-001072-15 EudraCT 2020-001228-32 ISRCTN89951424 NCT04324606 NCT04400838 NCT04444674 NCT04516746 NCT04540393 NCT04568031 PACTR202005681895696 PACTR202006922165132
NVX-COV2373	Novavax/Emergent Biosolutions/Praha Vaccines/Biofabri/Fujifilm Diosynth Biotechnologies/Fdb/Serum Institute Of India/Sk Bioscience/Takeda Pharmaceutical Company Limited/Agc Biologics/Polypeptide Group/Endo	India	Protein Subunit	Randomized, Quadruple Blind(Participant, Care Provider, Investigator, Outcomes Assessor), Parallel Placebo-controlled	2 (5 μg SARS-CoV-2 rS + 50 μg Matrix-M1 adjuvant (co-formulated))	0, 21days	IM	NCT04368988 NCT04533399 EudraCT 2020-004123-16 NCT04583995
mRNA 1273	Moderna/Niaid/Lonza/Catalent/Rovi/Medidata/Bioqual	USA	RNA	Randomized, Quadruple Blind(Participant, Care Provider, Investigator, Outcomes Assessor), Parallel Placebo-controlled	2 (100 μg)	0, 28days	IM	NCT04283461 NCT04405076 NCT04470427
BNT162	Biontech/Pfizer/Fosun Pharma/Rentschler Biopharma	Germany, China, USA	RNA	Randomized, Triple Blind(Participant, Care Provider, Investigator), Parallel Placebo Controlled	2 (10, 20, 30 μg BNT162b1 or BNT162b2, or 100μg BNT162b2)	0, 28days	IM	ChiCTR2000034825 EudraCT 2020-001038-36 NCT04368728 NCT04380701 NCT04523571 NCT04537949

## Inactivated Vaccines

When a new pathogen emerges, for example SARS-CoV, due to the lack of understanding of the pathogenesis and therefore lengthy time to development of efficacious therapeutics, the rapid and simple development of a vaccine against the emerging infectious disease is urgently needed. Therefore, the classic approach using inactivated, cell-culture based viruses is likely to be the fastest and easiest way for CoV vaccine development, as we have the experience of many commercial inactivated vaccines against other viral diseases. Inactivated vaccines may maintain the normal conformation of the S protein (71). Various studies have demonstrated that vaccines based on whole, inactivated SARS-CoV potently elicit considerable levels of neutralizing antibodies in animal models (72).

### SARS-CoV Inactivated Vaccines

Evaluations of a SARS-CoV whole virus vaccine double-inactivated with formalin and UV irradiation in ferrets and nonhuman primates showed protection against infection by SARS-CoV-specific T cell and neutralizing antibody responses. The immunogenic profile elicited by double-inactivated SARS-CoV vaccine might be not comparable to that generated by vaccine inactivated by just one approach (73). However, the challenged animals exhibited a Th2-type immunopathologic lung disease, whereas the pathologic changes seen in control groups lacked the eosinophil prominence (Tseng et al., 2012), which indicated that hypersensitivity to SARS-CoV components was induced. This may be called vaccine-associated disease enhancement (VADE) (74), which is similar to that seen with the RSV vaccine. Addition of an adjuvant in inactivated vaccines helped alleviate eosinophilic immune pathology in the lungs. Inactivated SARS-CoV vaccine with δ-inulin adjuvant (38) and ultraviolet inactivated SARS-CoV in toll-like receptor agonist reduced IL-4, IL-13, and eosinophil chemokines, resulting in the reduction of Th2 type eosinophilic pathology (39).

### MERS-CoV Inactivated Vaccines

The gamma-ray inactivated MERS-CoV vaccine with adjuvant of alum or MF59 induced high neutralizing antibodies but caused eosinophilic lung pathological changes in vaccinated animals (75, 76). Similarly, inactivated MERS-CoV vaccine appears to carry a hypersensitive-type lung pathology risk from MERS-CoV infection that is similar to that found with inactivated SARS-CoV vaccines from SARS-CoV infection (77). Formalin-inactivated MERS-CoV adjuvanted with alum and CpG induced high titers of anti-S IgG with neutralization reactivity >60%, and a stronger Th1/Th2 response in mice (75, 76). Both SARS-CoV and MERS-CoV vaccines inactivated by either gamma-ray or formalin induced immunopathologic lung disease in vaccinated animals, worthy of attention during the development of the inactivated SARS-CoV-2 vaccine.

### SARS-CoV-2 Inactivated Vaccines

Beta-propiolactone inactivated SARS-CoV-2 vaccines have been mainly developed in China. The vaccines developed by Wuhan Institute of Biological Products/Sinopharm, Beijing Institute of Biological Products/Sinopharm, and Sinovac/Instituto Butantan/Bio Pharma have been in clinical III trials. Unlike SARS-CoV and MERS-CoV, SARS-CoV-2 inactivated vaccines showed no evidence of immunopathologic changes in the lungs of vaccinated and SARS-CoV-2 challenged animals. On June 25, 2020, the vaccine candidate of Cansino/Military Academy of Sciences was approved as special drugs for the army by the Central Military Commission’s Health Bureau. On July 22, 2020, inactivated vaccine candidates of Sinopharm and Sinovac were approved for emergency use. On October 12, 2020, Sinopharm opened appointments for COVID-19 vaccination in Beijing and Wuhan. More than 70,000 people have made appointments for vaccination.

On April 12^th^, 2020, the inactivated vaccine from Wuhan Institute of Biological Products/Sinopharm was approved for clinical trials, which was the world’s first inactivated SARS-CoV-2vaccine that has received clinical trial approval. In phase I/II clinical trials, the vaccine induced high titers of antibodies in different doses, with the positive rate of neutralizing antibody reaching 100% and without adverse reactions (78). Up to September 8, 2020, a phase III clinical trial was ongoing in the UAE, Bahrain, Peru, Morocco, Argentina and other countries and regions, and this vaccine candidate is expected to be listed at the end of 2020 (https://www.echemi.com/cms/118167.html).

The inactivated vaccine from Sinovac also showed safety and effectiveness in rhesus monkeys, producing IgG and reducing virus titers and pathological changes in the lungs, without observable antibody-dependent enhancement of infection (79). On July 3, 2020, the inactivated vaccine from Sinopharm was approved for phase III clinical trial by Brazilian Health Regulatory Agency National Health Inspection Agency. On September 22, 2020, Sinovac started phase III Clinical Trials in Turkey (http://www.sinovac.com/?optionid=754&auto_id=911).

The third SARS-CoV-2 inactivated vaccine from Beijing Institute of Biological Products/Sinopharm started phase III trials in Argentina on September 16, 2020. This inactivated SARS-CoV-2 vaccine is prepared by inoculating African green monkey kidney cells (Vero cell) with the SARS-CoV-2 HB02 strain, culturing, harvesting, inactivating, clarifying, concentrating, purifying, and adding aluminum hydroxide adjuvant. The estimated study completion date is December 1, 2021.

The inactivated SARS-CoV-2 vaccine (Covaxin) developed by Indian Bharat Biotech is the sole inactivated vaccine which has entered phase II trials outside China. The animal experiments showed robust immune responses and protective efficacy, increasing SARS-CoV-2 specific IgG and neutralizing antibodies, reducing replication of the virus in the nasal cavity, throat, and lung tissues of monkeys. No evidence of pneumonia was observed by histopathological examination in vaccinated groups nor were adverse events seen in animals immunized with a two-dose vaccination regimen (http://mtw.so/5JtMTR).

## Live Attenuated Vaccines

With a long history of successful applications, such as the smallpox and polio vaccines, live attenuated vaccines are similar to natural infections with a wide range of natural viral antigen production over a long period of time and are often more immunogenic than non-replicating vaccines (71, 80).

CoV E protein induces endoplasmic reticulum stress (81) and inflammatory cytokine overexpression in host cells, causing lung tissue damage, edema, and progression to acute respiratory distress syndrome (ARDS). CoV lacking E protein has abnormal morphology and function due to assembly failure and maturation defects, and has been shown to inhibit the host cell’s stress response (2, 82).

### SARS-CoV Vaccines

SARS-CoV live attenuated vaccine with E gene deletion (SARS-CoV-ΔE) produced neutralizing antibodies and CD4^+^ with CD8^+^ T cell responses in mice and ferrets, and reduced inflammatory cell infiltration, edema, and cell destruction (41, 42). When the full-length E gene was deleted or its PDZ-binding motif (PBM) was mutated, revertant viruses either evolved a novel chimeric gene including PBM, or restored the sequence of the PBM in the E protein, respectively (43). Therefore, the modified virus with partial deletion of E gene without affecting PBM may be a live attenuated SARS-CoV vaccine candidate. Additionally, amino acid substitutions in the transmembrane domain (TMD) of E protein to eliminate the ion channel activity could result in virus attenuation, which alleviated pulmonary edema in infected mice. Another SARS-CoV vaccine candidate with simultaneous deletion of 8–12 amino acids in both C-termini of the E and nsp1 genes enhanced IFN responses and decreased viral titers in mice (43, 83).

Non-structural protein 1 (nsp1) (43), nsp16 (44), and nsp14 mutants of SARS-CoV and MERS-CoV have potential as live attenuated vaccines. SARS-CoV-ExoN (-) and MERS-CoV-ExoN (-) are both stable mutants providing immune protection in mice with significantly reduced fidelity and moderate pathogenicity (45, 46). Additionally, the combination of 2’-O-methyltransferase and ExoN mutations provided effective protection in the aged mice (84). Nsp10 is a major replication regulator in SARS-CoV and its deletion generated replication-deficient viruses by interfering and preventing the activation of nsp14 ExoN (85), which indicates a potential epitope for vaccine development.

### MERS-CoV Vaccines

CoV accessory proteins are implicated in the modulation of interferon signaling and proinflammatory cytokines (86). ORF3, 4a, 4b, and 5 are important for pathogenesis, and the MERS-CoV strains with combined deletion of the accessory genes 3, 4a, 4b, and 5 (rMERS-CoV-ΔORF3-5) significantly weakened virulence and reduced its inhibitory effects toward IFN, becoming a possible vaccine candidate (87). Additionally, MERS-CoV nsp16 mutants induced neutralizing antibodies and reduced viral titer in mice (88). Similarly, papain-like protease (Plpro) of SARS-CoV and MERS-CoV, encoded within nonstructural protein 3 (nsp3) of the replicase polyprotein, processed the viral replicase polyprotein and deubiquitinating (DUB) or deISGylating activity, and blocked upregulation of cytokines CCL5, IFN-β and CXCL10 in stimulated cells. Thus, mutation of Plpro of MERS-CoV inhibited the loss of IFN-β activation (89).

### SARS-CoV-2 Vaccines

There are four live attenuated COVID-19 candidate vaccines, developed by Mehmet Ali Aydinlar University & Acibadem Labmed Health Services A.S., Meissa Vaccines, Indian Immunologicals LTD & Griffith University, and Codagenix/Serum Institute of India, respectively. However, none of these vaccines have entered clinical trials. Among these vaccines, the vaccine candidate developed by Codagenix/Serum Institute of India is the earliest vaccine made in India and could induce strong immune responses. The vaccine was in the preclinical stage in April and is expect to enter clinical trials in September 2020.

The Bacillus Calmette-Guerin (BCG) vaccine was designed to protect against tuberculosis (TB). It boosts immunity by ‘training’ the immune system to respond to other subsequent infections with greater intensity. In order to find out whether it could reduce the risk of COVID-19 infection among healthcare staff and care home workers who are particularly vulnerable to coronavirus infection, this vaccine candidate is entering a phase III “BRACE” trial with up to 10,078 healthcare workers in hospitals in Australia as participants. In Netherlands, it is in phase IV trial and is enrolling 5,200 elders as participants (https://clinicaltrials.gov/ct2/results?cond=COVID&term=BCG+&cntry=&state=&city=&dist=;https://www.mcri.edu.au/news/could-bcg-vaccine-protect-against-covid-19-uk-recruitment-begins-0).

## Subunit Vaccines

Subunit vaccines are comprised of purified immunogenic proteins or peptides (90) derived from viruses. In contrast with traditional vaccines, subunit vaccines have less side effects and higher safety at the injection site. However, whether the immunological memory will be formed in the correct manner is not guaranteed. Therefore, adjuvants as well as vaccine delivery systems are needed to enhance immune responses (91).

### Spike/RBD-Targeted Subunit Vaccines

#### SARS-CoV S/RBD Vaccines

Most of CoV subunit vaccines focus on the S protein. S protein is the outermost localized protein responsible for receptor binding, especially its highly immunogenic receptor binding domain (RBD) (47), a critical region for receptor interaction (48).

In recent years, *Pichia pastoris* yeast have served as an expression system for producing a large number of modified proteins in the culture medium without animal-derived growth factors, thus widely applying to the pharmaceutical and vaccine industries. RBD 219N-1 protein, in which an N-linked glycosylated asparagine at the N-1 position of RBD219 has been deleted, expressed by *P. pastoris*, induced strong RBD-specific neutralizing antibody responses during pseudovirus and live SARS-CoV infections. Manufacture of recombinant RBD219-N1 protein was achieved with higher purity after optimizing the process (7, 8).

Recombinant fusion protein (RBD-Fc) containing 193-amino acid RBD (residues 318–510) and a human IgG1 Fc fragment with higher purification and stability as a vaccine candidate, enhanced antigen-presenting cell recognition by inducing strong neutralizing antibody and cellular immune responses and long-term protective effects in mice against SARS-CoV challenge (9–11).

#### MERS-CoV S/RBD Vaccines

The MERS-CoV spike protein forms a trimer, and its receptor-binding domain (RBD) serves as a vaccine target. RBD-Fd, a trimeric protein generated by fusing RBD with foldon trimerization motif (50). The outcomes indicated the potential of developing MERS subunit vaccines based on the trimeric RBD of MERS-CoV S protein. In a further study it was found that compared with Freund’s adjuvant, aluminum, monophosphorylate lipid A, and Montanite ISA 51, the combination of S377-588 protein fused with Fc of human IgG (S377-588-Fc) and MF59 adjuvant induced the highest titers of IgG, IgG1, and IgG2a subtypes and neutralizing antibodies after intranasal vaccination (49). Proline-substituted variants of MERS-CoV S2 domain retained S2 in the prefusion conformation, therefore producing a fully stable S trimer vaccine for broader and stronger neutralizing activity. Adjuvanted MERS-CoV S protein nanoparticles injected intramuscularly induced even higher levels of neutralizing antibodies (51).

‘Neutralizing immunogenicity index’ (NII) is a novel concept to evaluate the neutralizing immunogenicity of different epitopes on viral subunit vaccines. NII was used as a tool to identify epitopes with different neutralizing immunogenicity on a MERS-CoV-RBD-based vaccine. By application of this tool, subunit vaccines against MERS-CoV were rationally designed and found to significantly enhance the efficacy of the MERS-CoV RBD vaccine in protecting human-DPP4-transgenic mice from lethal MERS-CoV challenge (92). This methodology may guide the rational design of highly effective subunit vaccines to combat SARS-CoV-2.

#### SARS-CoV-2 Subunit Vaccines

B-cell and T-cell epitopes are highly conserved between SARS-CoV-2 and SARS-CoV. The vaccine against a conserved epitope may elicit cross-immune responses to mutant viruses (93). Analysis of T-cell and B-cell epitopes of SARS-CoV revealed that viral mutations mainly targeted epitopes that were highly expressed by MHC-I, while no mutations were found near RBD. In combination with other epitopes, recombinant SARS-CoV-2 S protein is a feasible vaccine candidate.

There are more than 60 subunit vaccines against SARS-CoV-2 under development, including RBD-trimer of S protein, S1, recombinant S proteins, N, M proteins, and others. One of them is in phase III clinical trial, four are in phase II trials, and seven are in phase I trials.

Novavax’s SARS-CoV-2 subunit vaccine candidate NVX-CoV2373 was based on Matrix-M -adjuvanted recombinant protein vaccine with nanoparticle technology using the Sf9 system. It has entered phase I/II clinical trials in May 2020, and has shown outstanding results so far without severe adverse events. It induced high titers of neutralizing antibodies and S protein specific IgG, as well as Th1 biased immune response while two doses of 5 μg of adjuvanted NVX-CoV2373 resulted in effective protection in non-human primate experiments, indicating the potential to protect humans (94) (https://ir.novavax.com/news-releases/news-release-details/novavax-initiates-phase-3-efficacy-trial-covid-19-vaccine-united). On September 24, 2020, Novavax initiated a phase III study of its vaccine candidate, expecting to enroll and immunize up to 10,000 individuals between 18 and 84 (inclusive) years of age in the UK. This is the fastest developed subunit vaccine against SARS-CoV-2.

In China, the SARS-CoV-2 RBD-dimer subunit vaccine by Anhui Zhifei Longcom/Institute of Microbiology, Chinese Academy of Sciences has entered a phase II clinical trial in July (https://clinicaltrials.gov/ct2/show/NCT04466085?term=NCT04466085&draw=2&rank=1). In Russia, on August 26, the Vector Institute registered a phase I/II trial for a subunit vaccine called EpiVacCorona. On October 14, Vladimir Putin announced that EpiVacCorona was granted regulatory approval to use in Russia. This is the second vaccine for limited use in Russia after the Gamelaya Institute’s Sputnik V vaccine. The third subunit vaccine which has entered phase I/II trials was developed by Sanofi/GSK. They launched a phase I/II clinical trial in September and plan to start a phase III trial in December. The fourth RBD subunit vaccine Soberana 1, which has entered phase I/II trials, was developed by Finlay Vaccine Institute in Havana, Cuba. It contains two extra ingredients: proteins from a bacteria and aluminum hydroxide as adjuvants (https://rpcec.sld.cu/ensayos/RPCEC00000332-Sp).

There are seven other SARS-CoV-2 subunit vaccines in phase I trials, developed by Medigen Vaccine Biologics Corp, Vaxine Pty Ltd, Clover Biopharmaceuticals and Gsk, Covaxx, University of Queensland, West China Hospital of China, and Adimmune Corporation. The safety and efficacy of these subunit vaccines should be verified in clinical trials.

### Nucleocapsid Proteins N as Immunological Target

During the SARS epidemic, it was found that the titer of neutralizing antibodies against S protein was significantly higher in deceased patients, while the anti-N protein antibody titer was lower during the early stages of infection (16, 17). In contrast, the anti-N antibody rose more rapidly in recovered patients (18, 95). The efficacy of anti-N antibody deserves further exploration.

In MERS-CoV, the B-cell, helper T-cell and cytotoxic T lymphocyte (CTL) epitopes were screened and mapped to the N protein, and are potential epitopes for vaccine candidates to elicit protective neutralizing antibodies and cellular immune responses against MERS-CoV (96). Along with the importance of T-cell-based cellular immunity, and escape of neutralizing antibodies against S protein of MERS-CoV due to its high mutation rate, N protein, rather than S protein, could be a suitable immunogen candidate with the potential to elicit both humoral and cell mediated immune responses (96). Currently the primary focus has been the spike protein. Whether the SARS-CoV-2 N protein is another potential immunological target for vaccines needs to be further verified.

## Virus-Like Particles (VLPs) and Bacteria-Like Particles (BLPs)

Virus-like particles (VLPs) are similar to intact virions in size and morphology. Without a viral genome, VLPs are unable to replicate or reverse mutate, suggesting better safety, especially for viruses that cause high morbidity and mortality. They may induce strong and broad humoral and cellular immune responses (20, 21, 97).

### SARS-CoV Vaccines

The S, M, and N proteins of the SARS-CoV are necessary and sufficient for pseudovirus assembly (25). Coexpression of SARS-CoV S protein and E, M and N proteins in mouse hepatitis virus (MHV) resulted in efficient production of MHV VLPs and protected the vaccinated mice from infection. Compared with the control groups, MHV VLPs adjuvated with alum induced high titers of neutralizing antibodies and reduced SARS-CoV titers as well as inflammation in the lung (22). Moreover, the influenza M1 protein is also a common core protein, suggesting the possibility of application to SARS-CoV VLP production. Researchers produced chimeric SARS VLPs (cVLPs) containing the spike protein of SARS and the matrix protein of influenza virus. VLPs with alum as an adjuvant induced significantly higher titers of neutralizing antibodies and protected mice against virus challenge, and led to lower virus titers after intramuscular immunization (98).

### MERS-CoV Vaccines

The avian influenza M1 was also used as a core protein to generate cVLPs containing modified S protein of MERS-CoV. This recombinant immunogenic cVLP significantly increased neutralizing antibodies and IgG against S protein of MERS-CoV in mice (23). Besides VLPs, a BLP vaccine candidate displaying the MERS-CoV RBD with GEL01 adjuvant also induced humoral, cellular, and local mucosal immune responses in the mouse model, especially in the intestinal tract, indicating its promise as a vaccine candidate (55). This BLP contains three lysin motif (LysM) motifs in an anchor protein combined with MERS-CoV RBD to form RBD-linker-PA3 (RLP_3_). Gram-positive enhancer matrix (GEM) particles were used as substrates to externally bind to the MERS-CoV RBD through a protein anchor. BLPs are a novel platform and have broad prospects in vaccine development.

### SARS-CoV-2 Vaccines

Fifteen VLP COVID-19 vaccines are in development. Among them, the vaccine developed by Medicago Inc. is the earliest and started in phase I trials in July 2020. The phase II/III trial is expected to start in November. This is a plant-derived VLP vaccine with GSK or Dynavax adjuvants. It uses the same platform as vaccine candidates for flu, rotavirus, norovirus, West Nile virus, and cancer.

Another VLP vaccine developed by SpyBiotech/Serum Institute of India entered phase I/II trials in September in Australia (https://www.spybiotech.com/news/-). This VLP displayed the RBD of SARS-CoV-2 S protein on the surface of Hepatitis B surface antigen (HBsAg) VLPs, which is safe and immunogenic and has been made in mass production.

The other VLP vaccines are still in preclinical phase. If the immunogenicity is proven, VLPs and BLPs are promising vaccine candidates for SARS-CoV-2 and other life-threatening viruses.

## Nucleic Acid Vaccines

Nucleic acid vaccines are genetic vaccines consisting only of DNA or RNA, which are taken up and translated into protein by host cells and elicit immune responses. Because they contain no viral coat, naked nucleic acids are not generally subject to preexisting immunity that can hamper the clinical efficacy of recombinant virus vaccines. In terms of higher safety and lower cost of production, nucleic acid vaccines have some major advantages over other types. Post-translational modifications under natural conditions are reproduced by the plasmid-encoded protein, retaining immunogenicity (99) and humoral and cellular immune-stimulating capabilities, simultaneously (24). Although there have been concerns about the safety of DNA vaccines in these early stages of development (100), it appears that viral genes integration into host genes through plasmid vectors is extremely rare (101).

### DNA Vaccines

#### SARS-CoV Vaccines

##### S Gene

SARS-CoV S DNA vaccines produced high levels of IgG against S protein (26) and CD4^+^ and CD8^+^ T cell responses (27). Furthermore, serum S protein-specific IgG1 and IgA in the respiratory tracts of mice were significantly elevated through PEI/pci-S complexes formed by polyethyleneimine (PEI) and SARS DNA vaccine, along with increases in IFN-γ, TNF-α, and IL-2 expression.

##### N Gene

Raghuwanshi et al. found that plasmid DNA loaded biotinylated chitosan nanoparticles for nasal immunization against N protein induced N protein-specific IgG, mucosal IgA, and IFN-γ expression in mice. When combined with CD40 monoclonal antibody, this vaccine induced higher antibody titers through intramuscular administration than by intranasal vaccination (28). Following intranasal delivery of naked pDNA, no mucosal and systemic immune responses were detected.

#### MERS-CoV Vaccines

There are several DNA vaccines of MERS-CoV under development: pVax1™ (GLS-5300), pVRC8400, and pcDNA3.1-S1 encoding MERS-CoV S1 subunit (56–60). These induced neutralizing antibodies and cellular immune responses in rhesus monkeys, camels, and mice. The IgG and specific cellular response levels of S1 subunit were higher than for S protein. A more balanced Th1/Th2 response avoided the potential safety issues of the S gene vaccines, i.e. the immunopathology and disease enhancement reported in SARS-CoV vaccine candidates (102, 103). The pVax1™ vaccine (GLS-5300) has completed phase I clinical trials. Most participants had three doses of vaccination, and anti-S1 subunit antibodies could still be detected after one year. The humoral and cellular immune responses of the subjects were similar to those recovering from natural infection of MERS-CoV. The vaccine was well tolerated, and no serious vaccine-related adverse events have been reported (57, 61).

Additionally, the MERS-CoV S protein vaccine supplemented with enhanced S1 subunit expression induced neutralizing antibodies and reduced disease severity in non-human primates (NHPs). Compared with pure protein and peptide vaccines, the combination of DNA and protein resulted in a more functional antibody library and stronger Th1 cell immune response (58).

#### SARS-CoV-2 Vaccines

Four DNA vaccine candidates have been studied in phase II clinical trials, including those developed by Inovio Pharmaceuticals (INO-4800), Zydus Cadila Healthcare Limited (ZYCOV-D), Osaka University (DNA plasmid+adjuvant), and Genexine consortium (GX-19), respectively. All these vaccine candidates are based on the spike protein, and have shown immunogenicity and protection in animals. Other DNA vaccines are still in preclinical stages.

INO-4800, a potential COVID-19 DNA vaccine candidate targeting SARS-CoV-2 S protein, induced effective humoral responses in mice and guinea pigs, and protected animals from lower respiratory disease (104). In early April, Inovio Pharmaceuticals started phase I clinical trials. 94% of participants developed the expected immune responses, including neutralizing antibodies and T cell immune responses without serious adverse reactions (http://ir.inovio.com/news-releases/news-releases-details/2020/INOVIO-Announces-Positive-Interim-Phase-1-Data-For-INO-4800-Vaccine-for-COVID-19/default.aspx). Phase IIa trials began in July 2020. However, Inovio announced a partial clinical hold for its phase II/III trial of on September 28, 2020. There were additional questions about INO-4800 reported to the US Food and Drug Administration (FDA), including its CELLECTRA^®^ 2000 delivery device. The phase II/III clinical trial will continue only when these questions have been satisfactorily answered (http://ir.inovio.com/news-releases/news-releases-details/2020/INOVIO-Reports-FDA-Partial-Clinical-Hold-for-Planned-Phase-2–3-Trial-of-COVID-19-Vaccine-Candidate-INO-4800/default.aspx).

In Korea, a DNA vaccine for COVID-19 named GX-19 developed by Genexine Inc. began phase I/IIa clinical trials in June 2020, expected to be completed by 2021 (https://www.bioworld.com/articles/435995-south-koreas-genexine-begins-phase-iiia-trials-for-covid-19-vaccine).

In Japan, a SARS-CoV-2 DNA vaccine developed by Osaka University/Anges/TAKARA BIO/Cytiva/Brickell BioTech is currently in phase I/II clinical studies (https://www.anges.co.jp/pdf_news/public/IGiJ94QWoV9U7EIYJybHY6SNv3BxVXRN.pdf). In India, a SARS-CoV-2 DNA vaccine given by intradermal route was developed by Cadila Healthcare Limited. It entered phase I trial in July 2020 and phase II in August 2020 (http://ctri.nic.in/Clinicaltrials/pmaindet2.php?trialid=45306&EncHid=&userName=vaccine).

### Conventional mRNA Vaccine

mRNA is a minimal and transient information carrier. It does not interact with the host genome, and is safe and can be manufactured rapidly. Any protein can be encoded and expressed by mRNA, which offers maximum flexibility with respect to the development of vaccines for infectious diseases and cancer as well as protein replacement therapies (105). The conventional mRNA vaccines translate the immunogens of interest from the input vaccine transcript.

While direct delivery into the cytosol would certainly enhance antigen expression, a lack of interaction with endosomal RNA receptors may severely weaken immunostimulation by the vaccine (105). Thus, suitable liposomes and complexing agents have been selected to enhance uptake by cells, improve delivery to the translation machinery in the cytoplasm, and prevent degradation of mRNA (105).

With the advantages of high efficiency, safety, low production cost, and the potential for rapid large-scale production, mRNA vaccines have become an attractive alternative to traditional vaccines, with a promising future. In animal models of infectious disease caused by influenza virus, Zika virus, rabies virus, the subcutaneous or intramuscular injection of liposome-encapsulated mRNA (106, 107) or a naked mRNA vaccine through subcutaneous or intranasal injection (108–111) induced effective immunity (106).

Several different mRNA vaccines exhibited high safety and tolerability in different stages of clinical trials. Nonetheless, the risk of an autoimmune response and/or promoting pathological thrombosis (112–115), or severe injection site or systemic reactions (107, 116) still exists in the application of extracellular RNA. Therefore, the safety of mRNA vaccines needs further evaluation. Moreover, the production of mRNA vaccines depends on a transcription system *in vitro*. When the production scale and speed cannot keep up with the speed of change in the epidemic, large-scale production applications remain challenging.

mRNA vaccines against SARS-CoV-2 developed by Moderna, BioNtech/Pfizer, Curevac, Arcturus, Academy of Military Sciences of China, Chulalongkorn University, and AstraZeneca/Shenzhen Kangtai have entered clinical trials. Among them, Moderna entered phase III trial in July, BioNtech/Pfizer started phase IIb/III trials in July, Curevac started IIa trials in September, Imperial College London started phase I/II trials in June, Arcturus started phase I/II trials in August, and Academy of Military Sciences of China started phase I trials in June. The others are still in development.

The mRNA-1273 vaccine candidate developed by Moderna is the most promising mRNA vaccine to date. This vaccine encodes the prefusion-stabilized spike protein of SARS-CoV-2, and induced S protein specific IgG antibodies in rhesus monkeys after the second vaccination. The vaccination induced type 1 helper T-cell (Th1)-biased CD4 T-cell responses and low or undetectable Th2 or CD8 T-cell responses in nonhuman primates. Compared to the inflammation of airways and adjacent alveolar interstitial found in the control group, animals in the mRNA-1273 group developed only mild inflammation and no viral RNA or antigen was detected in their lungs (117). In early July 2020, Moderna completed enrollment for both cohorts of its phase II study. The vaccine induced anti-SARS-CoV-2 immune responses in all participants, and no trial-limiting safety concerns were identified. Antibody titers were higher after the second vaccination (118). Later in July, Moderna launched a phase III clinical trial (https://investors.modernatx.com/news-releases/news-release-details/moderna-announces-phase-3-cove-study-mrna-vaccine-against-covid).

Another hopeful mRNA vaccine BNT162 was developed by BioTech/Fosun Pharma/Pfizer. Two mRNA candidate vaccines were evaluated in the phase I portion of the trial in the United States: one was BNT162b1, encoding a secreted trimerized SARS-CoV-2 receptor–binding domain, and the other was BNT162b2, encoding a membrane-anchored SARS-CoV-2 full-length spike, which was stabilized in the prefusion conformation. BNT162b2 was associated with a lower incidence and severity of systemic reactions than BNT162b1, particularly in older adults (119). In both younger and older adults, the two vaccine candidates elicited similar dose-dependent SARS-CoV-2–neutralizing geometric mean titers (119). A phase IIb/III trial was launched in July 2020. On November 9, they announced that the vaccine candidate BNT162b2 was found to be more than 90% effective in preventing COVID-19 in participants without evidence of prior SARS-CoV-2 infection in the first interim efficacy analysis from the phase III clinical study (https://www.pfizer.com/news/press-release/press-release-detail/pfizer-and-biontech-announce-vaccine-candidate-against). The trial is continuing to enroll and is expected to continue through the final analysis when a total of 164 confirmed COVID-19 cases have accrued.

CVnCoV is a mRNA vaccine developed by Curevac. They launched a phase IIa clinical trial on September 29, 2020. Curevac reported preclinical trial data through October 23, 2020. After the second vaccination, titers of neutralizing antibodies and IgG were significantly higher and more lasting. The IgG2a/IgG1 ratios showed a balanced Th1/Th2 profile, and the vaccine appeared to avoid vaccine-induced disease enhancement (https://www.curevac.com/en/2020/10/23/curevac-reports-positive-preclinical-data-for-its-covid-19-vaccine-candidate-cvncov/).

The other mRNA vaccine candidate that entered human phase I/II clinical trials in June was developed by Imperial College London (https://www.imperial.ac.uk/news/198314/imperial-begin-first-human-trials-covid-19). In China, the first COVID-19 mRNA vaccine approved for clinical trials was ARCoV, developed by the People’s Liberation Army (PLA) Academy of Military Sciences, Suzhou Abogen Biosciences, and Walvax Biotechnology Co., Ltd. Their study showed that the COVID-19 mRNA vaccine not only induced high levels of neutralizing antibodies in mice and crab-eating macaques but also induced protective T cell immune responses (120). ARCoV is currently being evaluated in phase 1 clinical trials. Other mRNA vaccine candidates showed immunogenicity by eliciting potent neutralizing antibodies in mice and/or NHPs (121, 122).

### Self-Replicating RNA Vaccine (saRNA)

Self-amplifying RNA (saRNA) is derived from an alphavirus genome, which encodes the alphaviral replicase and a gene of interest. It amplifies sub-genomic RNA carrying the antigen of interest, resulting in the amplification of transcripts bearing the antigen by several orders of magnitude over the initial dose (123). saRNA is a highly efficient platform for SARS-CoV-2 vaccine development.

Currently, among several saRNA vaccine candidates against SRAS-CoV-2, LUNAR-COV19 developed by Arcturus Therapeutics and Duke-NUS Medical School is the sole one in clinical trials. This vaccine encodes SARS-CoV-2 full length S protein and requires a much smaller dose than the conventional mRNA vaccine, ~ 50 to 100 times less, which would greatly lower the cost per dose. Mice vaccinated with a single dose of LUNAR-COV19 induced stronger T cell responses and significantly higher levels of S protein specific IgG lasting for 50 days after vaccination, as well as robust neutralizing antibodies. Similarly, Th1 biased immune responses were shown in this study, and LUNAR-COV19 also protected mice from SARS-CoV-2 lethal challenge and even measurable infection (124).

## Viral Vector Vaccines

Viral vector vaccines can effectively introduce genes encoding viral antigens into host cells. The infected cells produce and release immunogenic antigens within a certain period after vaccination (80). Subunit vaccines and protein-induced immune responses are usually short-lived, and consequently multiple injections are usually required to induce and maintain a systemic immune response. In contrast, nonattenuated viral vectors can invade cells naturally, thus activate the immune system and induce stronger humoral and cellular immune responses. Several viral vectors for CoV vaccines have been developed, such as adenovirus (AdV), modified vaccinia virus Ankara (MVA), measles virus (MV), Venezuelan equine encephalitis virus (VEE), vesicular stomatitis virus (VSV), Newcastle disease virus (NDV), rabies virus (RV), RSV, and others (125, 126). These virus-based vectors provide innovative directions and routes for CoV and other virus vaccine research and development. Through November 3, 2020, 18 replicating viral vector vaccines and 26 non-replicating viral vector vaccines were under development for COVID-19. The former are designed mainly on measles virus, VSV, influenza virus, avian paramyxovirus, and NDV. The latter are based mainly on human adenovirus types 5 or 26, chimpanzee adenovirus, Parainfluenza Virus 5(PIV5), influenza virus, AAV, and MVA.

### Adenovirus Vectored Vaccines

#### SARS-CoV Vaccines

Human adenovirus type 5 (HAdV-5) vectored vaccines have been extensively developed (33, 34). HAdVs can effectively induce mucosal immune responses, and have been widely studied for their wide host range, strong infectivity, high protein expression, and high safety when imbued with a replication defect. Compared with intramuscular injection, both intranasal and sublingual administration of recombinant adenoviruses encoding SARS-CoV spike protein elicited stronger CD8^+^ cell response and higher levels of neutralizing antibodies and IgA without VADE (35).

#### MERS-CoV Vaccines

A single injection of the MERS-CoV S protein-coding HAdV-5 or HAdV-41 vectored vaccines elicited mucosal and systemic immunity in mice (33, 34). When boosted with S nanoparticles, the vaccine induced S-specific IgG neutralizing antibodies, as well as Th1 and Th2 cell immune responses to protect adenoviral hDPP4-transducted mice from MERS-CoV challenge (66).

However, it is known that pre-existing immunity to prevalent adenovirus serotypes can inhibit the efficacy of adenovirus-vectored vaccines (127, 128). Therefore, exploring other AdV types as vectors has become a possible solution. The simian adenovirus is an alternative choice for a vector because of minimal pre-existing immunity in humans, except for the Africans (129). The replication-deficient chimpanzee adenoviruses ChAdOx1 and AdC68 (66, 130) expressing MERS-CoV proteins significantly reduced clinical signs in camels (67), induced sustained and high levels of neutralizing antibodies and T cell responses in mice (70), and protected mice against lethal challenge (68). A phase II clinical trial is now underway.

#### SARS-CoV-2 Vaccines

The vaccine candidate AZD1222 is based on ChAdOx1 (a MERS-CoV vaccine mentioned above), and utilizes a replication-deficient chimpanzee adenovirus vector. This entered phase II/III clinical trials in the UK and India in May 2020, and phase III trials in Brazil, South Africa, and the United States. It was developed by Consortium of the Jenner Institute/Astrazeneca/University of Oxford. In their phase I/II, single-blind, randomized controlled trial, spike-specific T-cell responses peaked on day 14, and anti-spike IgG responses rose by day 28, while neutralizing antibodies were shown in all participants after a booster dose. Adverse reactions were significantly reduced by use of prophylactic paracetamol (131).

However, in September, the phase III trial was halted because one volunteer developed transverse myelitis. On October 21, it was reported that that a volunteer in Brazil who once worked in a hospital and had received a dose of placebo in the trial, died of COVID-19 (https://www.nytimes.com/live/2020/10/21/world/covid-19-coronavirus-updates/a-vaccine-trial-volunteer-in-brazil-has-died-but-health-authorities-say-the-vaccine-was-not-to-blame). However the trial was soon restarted. The safety of the chimpanzee adenovirus vectored SARS-CoV-2 vaccine will be closely monitored going forward.

In China, human adenovirus type 5 vectored COVID-19 vaccine (Ad5-nCoV) developed by Cansino Biologics/Beijing Institute of Biotechnology entered phase I trials in early March and phase II trials in April. phase III trials began in August, 2020. The vaccine was approved for China military use on June 25, 2020, which was the first approved COVID-19 vaccine for limited use. Most adverse reactions reported in all dose groups were mild or moderate in severity. No serious adverse event was noted within 28 days post-vaccination. The vaccine showed immunogenicity and tolerance 28 days after the first inoculation. Neutralizing antibodies peaked at day 28 post-vaccination, and specific T-cell response peaked at day 14 (132). In a phase II clinical trial in 508 participants, the Ad5-vectored COVID-19 vaccine given at 5 × 10^10^ viral particles showed safety and induced significant immune responses in the majority of recipients after a single immunization (133). The vaccine will be studied in a phase III trial in Saudi Arabia and Russia (https://www.arabnews.com/node/1717041/saudi-arabia; https://www.sohu.com/a/414125695_115479). Another HAdV-5 vectored COVID-19 vaccine candidate developed by a Chinese research group also conferred protection from SARS-CoV-2 challenge in rhesus macaques with a single vaccination either intramuscularly or intranasally (134).

On October 8th, China officially joined the COVAX, a global COVID-19 vaccine allocation plan co-led by the World Health Organization (WHO) that aims to help purchase and fairly distribute COVID-19 vaccines (https://www.weforum.org/agenda/2020/09/covax-who-cepi-gavi-covid-19-coronavirus-vaccines-distribution/). CanSino Biologics announced a supply agreement of 35 million doses of COVID-19 vaccine for Mexico from the end of 2020 through the year 2021. Compared with other vaccines developed by Pfizer, AstraZeneca, Covax, the Ad5-nCoV vaccine of CanSino Biologics is the only single-dose regime candidate (http://www.cansinotech.com/html/1///179/180/556.html).

The other approved COVID-19 vaccine is developed by Gamaleya Research Institute of Russia. The first dose contains HAdV-26 vectored vaccine, based on an uncommon adenovirus type. The booster dose is composed of HAdV-5 vectored vaccine, similar to the one being developed by CanSino, China. The phase I trial began in June 2020. Phase 1/2 non-randomized studies of a heterologous prime-boost COVID-19 vaccine based on rAd26-S and rAd5-S showed that the vaccine was safe and well tolerated. Cellular immunity, neutralizing antibodies, and RBD specific IgG were detected in all participants, and no severe adverse reactions were reported after vaccination (135). Named Sputnik-V, this vaccine received early approval for use in Russia in August 2020 without completing a phase III trial (https://www.nature.com/articles/d41586-020-02386-2). On October 14, 2020, Russia granted regulatory approval to a second COVID-19 vaccine, even though the vaccine had yet to begin large scale phase-III trials. This was only two months after approval of their first vaccine. Experts expressed caution due to a lack of safety and efficacy data (https://www.webmd.com/lung/news/20200908/russia-begins-rollout-of-covid-19-vaccine). Due to the incomplete completion of phase III clinical trials, the safety of these vaccine candidates is expected to be evaluated with special scrutiny (https://www.usnews.com/news/world/articles/2020-08-20/un-discussions-with-russia-on-covid-19-vaccine-under-way).

Another HAdV-26 vectored vaccine is developed by Janssen Pharmaceutical Companies of Johnson & Johnson. In the phase I/II clinical trial, the vaccine JNJ-78436735 induced robust humoral and cellular immune responses in middle-age adults and the elderly (136). On September 23, 2020, Johnson & Johnson announced the launch of its large-scale, pivotal, multi-country phase III trial(named ENSEMBLE) for its COVID-19 vaccine candidate. This study enrolled up to 60,000 adults 18 years old and older, including participants over 60 years old, and those both with and without comorbidities associated with an increased risk for progression to severe COVID-19 (https://www.jnj.com/johnson-johnson-initiates-pivotal-global-phase-3-clinical-trial-of-janssens-covid-19-vaccine-candidate).

It is optimized that the type of adenovirus vectors used in the boost dose is different from that in the initial immunization. In theory, the alternate use of human and simian adenovirus vectors in the immunization steps is better than the use of the same type of adenovirus vectors. Additionally, the grouping of participants with high and low levels of pre-existing immunity against the adenovirus vectors should be considered.

### MVA Vectored Vaccines

#### SARS-CoV Vaccines

Modified vaccinia virus Ankara (MVA) is replication-defective and used for viral antigen expression in mammalian cells. MVA stimulates inflammatory cytokines and chemokines and migration of lymphocytes and monocytes, making it advantageous in vaccine applications. MVA-SARS-CoV produced effective neutralizing antibodies with high immunogenicity in mice, rabbits, and monkeys, and protected against challenge in mice (29–32).

#### MERS-CoV Vaccines

MVA-MERS-S protein vaccines induced neutralizing antibodies and CD8 ^+^ T cell responses (62) and prevented tissue damage in AdV-hDPP4 transgenic mice (63). Likewise, intramuscular administration of MVA-MERS-CoV induced neutralizing antibodies in dromedary camels and limited virus replication, resulting in effective immune protection (64).

#### SARS-CoV-2 Vaccines

There are five MVA vectored SARS-CoV-2 vaccines under development. Among them, the spike gene vaccine developed by German Center for Infection Research is registered in a phase I trial. The vaccine is expected to be ready for approval by the end of 2021.

### Other Virus Vector Vaccines

#### SARS-CoV Vaccines

Although the immunity of Measles virus (MV) vector among humans may be an obstacle for its application, the SARS-CoV S protein expressed by the MV vector induced high neutralizing antibodies and Th1 cell immune responses in susceptible mice, along with effective protection from challenge. Similarly, a recombinant Venezuelan equine encephalitis virus (VEE)-SARS-CoV vaccine elicited high IgG titers in mice while retaining wild-type VEE replication ability (36, 37, 137). The use of MV and VEE as viral vectors for CoV vaccines may yet have potential.

#### MERS-CoV Vaccines

A single dose of parainfluenza virus 5 (PIV5)-based vaccine expressing the MERS-CoV S protein induced neutralizing antibodies and robust T cell responses in hDPP4 mice. A single-dose intranasal immunization brought stronger protection than single-dose intramuscular immunization. Mice immunized with PIV5-MERS-S protein developed greater mononuclear cell infiltration and less pathological changes in infected lungs (including edema, hyaline membranes, necrotic cellular debris, etc.), as well as complete protection against a lethal challenge against MERS-CoV and lower viral titers. Compared to inactivated MERS-CoV, histopathological changes in lungs such as hyaline membrane formation and hypersensitivity-type response with perivascular eosinophilic infiltration were milder (138).

#### SARS-CoV-2 Vaccines

In 2019, researchers at the University of Hong Kong and Xiamen University developed a nasal-spray vaccine for the flu based on a genetically weakened influenza virus. Earlier this year, they engineered the same vaccine to produce coronavirus spike protein. On September 9, they received approval to start clinical trials (https://www.hku.hk/press/news_detail_21583.html).

Merck has developed a SARS-CoV-2 vaccine originally developed at Institute Pasteur using a weakened measles virus that carries the coronavirus spike gene. They launched phase I trials in August (https://clinicaltrials.gov/ct2/show/NCT04497298?term=vaccine&cond=covid-19&draw=2&rank=1).

In addition, the intranasal vaccine candidate (MV-012-968) expressing SARS-CoV-2 S protein based on an RSV vector was developed by Meissa. It showed robust immune responses in rats and in healthy adults, and is currently in phase I trials in healthy adults and young children (https://www.biospace.com/article/another-covid-19-vaccine-joins-the-race-this-time-it-s-a-live-weakened-virus/?tdsourcetag=s_pctim_aiomsg).

A SARS-CoV-2 S gene vaccine based on adeno-associated virus was developed by the Massachusetts Eye and Ear, Massachusetts General Hospital and the University of Pennsylvania. Phase I trials are set to begin in late 2020.

## Antibody-Dependent Enhancement (ADE)

ADE is an adverse reaction in which non-neutralizing antibodies produced following virus infection or a vaccination enhance the infectivity of a subsequent virus infection (139). It is a mechanism found to play a role in infection by dengue viruses, HIV, influenza virus, Ebola virus, feline coronavirus, and in SARS-CoV, which facilitates the infection of host target cells by anti-viral humoral immune responses. ADE can be mediated by antibody Fc receptor-associated internalization of the virus, resulting in greater viral replication and cytokine release in the presence of virus-specific antibodies (74). ADE may occur especially when the antibody levels are relatively lower.

It was reported that SARS-CoV used ADE to enhance the infectivity of human promonocytes. Increased TNF-α, IL-4, and IL-6 were detected in human promonocytes isolated from a leukemia patient (HL-CZ cells) infected with SARS-CoV, and treatment with highly diluted anti-sera against SARS-CoV was associated with higher levels of virus infection in cells and increased cytopathic effect (CPE) (140). Moreover, S protein specific-IgG may have promoted proinflammatory cytokine production through FcγRI and/or FcγRIIA, suggesting a potential role of FcγRs for the postulated reprogramming of alternatively activated macrophages. Blockade of FcγRs reduced proinflammatory cytokine production and lung injury (19).

To date, no ADE has been observed in MERS-CoV and SARS-CoV-2. However, due to the taxonomic and structural similarities between SARS-CoV, MERS-CoV, and SARS-COV-2, ADE is an issue that should be considered seriously in designing MERS-CoV and SARS-CoV-2 vaccines, particularly those with a full-length S protein. Neutralizing epitopes could elicit a more robust protective immunity but less or no ADE side-effects. Recent studies have found that MERS-CoV vaccine candidates based on a shorter S1 domain or shorter RBD induce stronger immune responses than those based on the full-length S protein (48, 141). Whether the ADE is common during all coronavirus infections needs further study and verification.

## Discussion

From the SARS epidemic 17 years ago to the MERS-CoV epidemic in 2012, and the COVID-19 pandemic caused by the newly emerged SARS-CoV-2 in December 2019, the threat of future severe acute respiratory diseases due to the CoV family cannot be underestimated. Clinical trials of SARS-CoV vaccines were terminated due to the disappearance of SARS-CoV and the lack of potential patients. The MERS-CoV vaccine completed only phase I clinical trials in humans. The shared experiences in developing SARS-CoV and MERS-CoV vaccines may provide a reference for COVID-19 vaccine development.

Currently, the many different SARS-CoV-2 vaccines are in different stages of development around the world. Different types being tested include recombinant protein subunit vaccines, nucleic acid vaccines, viral vector vaccines, inactivated viruses, and live attenuated vaccines. Among the 212 vaccines being developed, three inactivated vaccines, 4 non-replicating viral vector vaccines, two protein subunit vaccines and two RNA vaccines have entered phase III clinical trials. One human adenovirus vector vaccine and three inactivated vaccines have been approved for limited use in China. In Russia, one adenovirus vector vaccine and one peptide vaccine have been approved for early use. The mRNA vaccine of Moderna, HAdV-5 vector vaccine of CanSino Biologics, inactivated vaccines of Sinopharm, Wuhan Institute of Biological Products, and Sinovac Biotech have all entered phase III clinical trials. The mRNA vaccine of BioNTech, Germany, and the Chimpanzee adenovirus vector vaccine of AstraZeneca and the University of Oxford have entered phase II/III clinical trials (**Table 3**). Together, these represent the most promising and earliest candidate vaccines against COVID-19.

There remain numerous unknowns for all the current vaccine candidates. For examples, will the vaccine provide effective protection in immune deficient or dysfunctional patients and the elderly, and for how long will it provide immunity? Recently, HKU researchers have confirmed the world’s first case of reinfection by the SARS-CoV-2 (https://www.sciencenews.org/article/coronavirus-covid-19-first-case-reinfection-man-hong-kong), in which reinfection occurred after just a few months from the first infection. SARS-CoV-2 may persist as a source of human infections as is the case for other common-cold associated human coronaviruses, even if patients have acquired some level of immunity *via* natural infection. If the immunity to SARS-CoV-2 can disappear after natural infection, vaccination should also be considered for those persons with prior infection. Additionally, the combined use of nucleic acid vaccines, subunit vaccines, inactivated vaccines, and viral vector vaccines with nanoparticle technology or adjuvants, and multiple vaccinations of COVID-19 should be considered if immunity wanes over time.

There is consensus that the engagement of the S1 RBD with its receptor destabilizes the trimer (prefusion state), triggering the shedding of the S1 units, which allows a remarkable conformational change in the spike from a large club-shaped structure into a thin and long nail-like structure (postfusion state). β-propiolactone is the chemical inactivating agent successfully used in rabies and other vaccines, and β-propiolactone-treated SARS-CoV-2 viruses exhibit most of their spikes in the postfusion conformation (142). Most COVID-19 vaccine candidates rely on the S protein as their antigen, since this is the primary exposed protein on the surface of the SARS-CoV-2 viral particle, and β-propiolactone is used as the inactivation reagent. However, there is the unfortunate example of the formalin-inactivated respiratory syncytial virus (FI-RSV) vaccine trial of the 1960s, which led to enhancement of disease symptoms in vaccinated children after natural exposure to RSV, with two fatal cases (143). Structural studies revealed that one contributing factor to the vaccine failure was that the prefusion state of the RSV spike was absent and the postfusion state was primarily represented in the FI-RSV vaccine formula (144). Therefore, the inactivated SARS-CoV-2 vaccines may not be the safest, and there is need to confirm the S protein state.

It is well known that RNA viruses have much higher mutation rates than DNA viruses. More and more mutations in the spike protein of SARS-CoV-2 are being continuously reported (145, 146), e.g., the most dominant variant D614G in spike protein (146, 147) and V367F in RBD (145), which may increase viral infectivity. As the spike protein of coronaviruses is a major target for vaccines, neutralizing antibodies, and viral entry inhibitors, spike protein mutations in circulating viral strains may affect the effectiveness of a vaccine. Therefore, it is very important to determine whether the neutralizing capacity of vaccine-induced neutralizing antibodies remain unchanged in clinical trials over time.

While clinical treatment strategies have been optimized to save lives and improve prognosis, a safe and effective vaccine would have far-reaching public health significance for controlling and stopping the COVID-19 pandemic. Clinical trials are indispensable to determine the safety and effectiveness of COVID-19 vaccines, and will also need to evolve to include children, the elderly, pregnant women, and people with underlying medical conditions.

## Author Contributions

QZ conceptualized the idea for the review. JZhao, SZ, JO, JZhang, WL, WG, XW, and YY performed the literature search, analyzed cited references, and wrote the original article. WZ, JW, JC and QZ critically revised the article. All authors contributed to the article and approved the submitted version.

## Funding

This work was supported by grants from the National Key Research and Development Program of China (2018YFE0204503) and Natural Science Foundation of Guangdong Province (2018B030312010), as well as the Guangzhou Healthcare Collaborative Innovation Major Project (201803040004 and 201803040007).

## Conflict of Interest

The authors declare that the research was conducted in the absence of any commercial or financial relationships that could be construed as a potential conflict of interest.
